# Osteoarticular Coccidioidomycosis in California: A Single-Center Experience

**DOI:** 10.1093/ofid/ofag103

**Published:** 2026-02-24

**Authors:** Radhika Arya, Melissa Dzinoreva, Traci Shiu, Elizabeth Thottacherry, Amy Chang, Jenny Aronson, Shanthi Kappagoda, Daisuke Furukawa

**Affiliations:** Division of Infectious Diseases and Geographic Medicine, Stanford University School of Medicine, Stanford, California, USA; Stanford University School of Medicine, Stanford University, Stanford, California, USA; Stritch School of Medicine, Loyola University Chicago, Maywood, Illinois, USA; Division of Infectious Diseases and Geographic Medicine, Stanford University School of Medicine, Stanford, California, USA; Division of Infectious Diseases and Geographic Medicine, Stanford University School of Medicine, Stanford, California, USA; Division of Infectious Diseases and Geographic Medicine, Stanford University School of Medicine, Stanford, California, USA; Division of Infectious Diseases and Geographic Medicine, Stanford University School of Medicine, Stanford, California, USA; Division of Infectious Diseases and Geographic Medicine, Stanford University School of Medicine, Stanford, California, USA

**Keywords:** coccidioidomycosis, osteoarticular infection

## Abstract

**Background:**

Osteoarticular involvement in coccidioidomycosis is an uncommon manifestation leading to significant morbidity, but evidence surrounding it is limited. We aimed to describe the clinical characteristics of osteoarticular coccidioidomycosis and identify factors associated with treatment failure.

**Methods:**

We performed a retrospective chart review of adults age ≥18 years hospitalized with confirmed osteoarticular coccidioidomycosis at an academic tertiary care center between 2004 and 2021. We extracted demographic, clinical, microbiologic, treatment, and outcomes data. Univariable regression analysis was used to identify risk factors of disease progression or relapse.

**Results:**

Thirty-two patients were reviewed, of whom 29 (91%) were male, with a median age (interquartile range [IQR]) of 46.8 (35.1–65.6) years and a median time of follow-up (IQR) of 84 (47–127) months. The most common sites of infection were spine (n = 15, 47%) and knee (n = 9, 28%). Itraconazole was the most common antifungal used (n = 16, 50%), followed by posaconazole (n = 8, 25%), and surgery was performed in 24 (75%) patients. The median treatment duration (IQR) was 45.0 (13.3–66.7) months, with 26 (81%) patients remaining on antifungals through the last day of follow-up. Fifteen (47%) patients experienced progression and/or relapse. Knee (odds ratio [OR], 18.29; 95% CI, 91–175.35) and multisite infections (OR, 6.56; 95% CI, 1.10–39.32) were associated with disease progression and/or relapse, while spine infection was associated with lower rates of progression and/or relapse (OR, 0.20; 95% CI, 0.44–0.90).

**Conclusions:**

Patients with osteoarticular coccidioidomycosis required prolonged therapy with a substantial risk of disease progression or relapse. Knee and multisite infections were associated with poorer outcomes. Larger studies are needed to validate these findings and optimize treatment strategies.

## Teaser

Patients with osteoarticular coccidioidomycosis had high rates of disease progression and relapse despite prolonged antifungal treatment and surgical interventions. Knee and multisite infections were associated with higher risk of treatment failure while spinal infections had lower risk of failure.

Coccidioidomycosis, caused by *Coccidioides immitis* and *Coccidioides posadasii*, is an endemic fungal infection with high prevalence in the Southwestern United States and Northern Mexico. Nearly 20 000 cases are reported annually in the United States, with the majority presenting as pulmonary disease [1]. Disseminated infection occurs in ∼1% of cases, and osteoarticular involvement, while relatively uncommon, represents a significant cause of morbidity due to its chronicity, diagnostic challenges, and high relapse rates [2, 3].

Bone and joint disease due to *Coccidioides* most commonly involves the axial skeleton, especially the spine, followed by large joints such as the knee. Skeletal disease may present with pain, swelling, and, in severe cases, neurological deficits secondary to spinal involvement [2]. Current treatment paradigms emphasize the use of azole antifungals as first-line therapy for most patients, with amphotericin B reserved for severe or refractory cases [2, 4, 5]. Despite antifungal therapy being the mainstay of treatment, surgery is frequently performed, and relapses remain a challenge [2, 3, 4, 6].

Though osteoarticular coccidioidomycosis is a well-recognized disease entity, its literature base is limited to case reports and small case series [2, 4, 7, 8]. As such, there remain significant gaps in knowledge regarding optimal antifungal agents, treatment duration, role of surgery, and risk factors for treatment failure. The current treatment guideline is reflective of this as its recommendations are largely based on low level of evidence and expert opinion [5]. To fill this gap, our study aimed to characterize demographic and clinical patterns of osteoarticular coccidioidomycosis, describe treatment approaches, and identify risk factors associated with disease progression or relapse. To our knowledge, this is one of the largest single-center studies in adults, and no prior studies have evaluated risk factors of treatment failure specifically for osteoarticular coccidioidomycosis.

## METHODS

We conducted a retrospective chart review of adult patients (≥18 years) admitted with confirmed osteoarticular coccidioidomycosis between 2004 and 2021 at an academic tertiary care hospital in California. Patients were queried from our institution's clinical data warehouse through the STAnford medicine Research data Repository (STARR) platform using International Classification of Diseases (ICD) codes from both the ninth and tenth revisions (ICD-9 and ICD-10). Inclusion criteria consisted of patients ≥18 years of age with microbiologic evidence of coccidioidomycosis (culture, histopathology, antigen, sequencing, or serology) and radiographic or intraoperative findings consistent with skeletal infection. Exclusion criteria were patients without osteoarticular involvement, those lacking available clinical records, patients whose primary management occurred at outside hospitals, or patients who had <1 year of follow-up from initiation of antifungal treatment.

Data abstracted from the electronic medical record included demographics, comorbidities, history of pulmonary or prior coccidioidomycosis, site(s) of skeletal infection, extraskeletal involvement, laboratory values, radiographic findings, antifungal regimens, surgical interventions, and clinical outcomes. All data were recorded in REDCap.

The primary outcome was disease progression and/or relapse. Progression was defined as radiographic or symptomatic worsening in addition to either a 4-fold increase in *Coccidioides* complement fixation (CF) titer or subsequent tissue confirmation of residual infection on culture or pathology while on antifungal treatment. Relapse was defined as those who met the same criteria as progression after stopping antifungal treatment. Outcome at the last documented infectious diseases follow-up visit was defined as either (1) off antifungal, (2) on antifungal for treatment of active infection, defined as positive CF titer or ongoing symptomatic or radiographic disease, or (3) on antifungal for suppression, defined as treatment in the setting of negative serologic, symptomatic, or radiographic evidence of disease. Manual chart review was conducted primarily by 3 trained reviewers (R.A., M.D., T.S.), and patient outcomes were also evaluated by a fourth reviewer (D.F.) to ensure accuracy.

Categorical variables were summarized by frequency and proportion, and continuous variables were summarized by median and interquartile range (IQR). We compared variables between those who experienced progression or relapse and those who did not experience progression or relapse. For group comparisons, the Fisher exact test was used for categorical variables, and the Wilcoxon rank-sum test was used for continuous variables. For variables with *P* < .10 in the bivariate analysis, univariable regression analysis was additionally performed to identify risk factors for progression or relapse. Odds ratios (ORs) with 95% CIs were calculated, and a *P* value <.05 was considered statistically significant. The institutional review board at our institution approved this study.

## RESULTS

### Patient Characteristics

Three hundred eighty-nine patient records were initially queried with ICD codes. After screening for inclusion and exclusion criteria, a total of 32 patients with osteoarticular coccidioidomycosis were included (Table 1). The median age (IQR) was 46.8 (35.1–65.6) years, and 29 (91%) were male. Race distribution included Hispanic (n = 12, 38%), non-Hispanic White (n = 10, 31%), and other racial/ethnic groups (n = 10, 31%). Comorbidities were common: 10 (31%) had diabetes mellitus, 15 (47%) had a prior known diagnosis of coccidioidomycosis, and 4 (13%) were immunosuppressed due to corticosteroids, transplant, or other immunosuppressive medications. Diagnostic tests performed included serology (n = 28, 88%), culture (n = 23, 72%), histopathology (n = 14, 44%), or sequencing/antigen (n = 3, 9%). Lung involvement was the most common extraskeletal site (n = 19, 59%), followed by skin/soft tissue (n = 8, 25%) and meningitis (n = 2, 6%) at time of presentation. The majority presented with musculoskeletal pain (n = 28, 88%) or joint swelling (n = 11, 34%). The median duration of symptoms (IQR) before antifungal initiation was 5 (2–12) months.

**Table 1.** Baseline Characteristics of Patients With Osteoarticular Coccidioidomycosis

	Total n = 32, No. (%)
Age, median (IQR), y	46.8 (35.1–65.6)
Male	29 (91)
Race	
Hispanic	12 (38)
Non-Hispanic White	10 (31)
Non-Hispanic Black	5 (16)
Asian	2 (6)
Other	3 (9)
Comorbid conditions	
Diabetes mellitus	10 (31)
Solid tumor	1 (3)
Prior known diagnosis of coccidiodomycosis	15 (47)
Immunosuppressed	4 (13)
Involved bone/joint	
Spine	15 (47)
Wrist/hand	3 (9)
Elbow	1 (3)
Shoulder	5 (16)
Hip	3 (9)
Knee	9 (28)
Foot/ankle	2 (6)
Long bone lower extremity	4 (13)
Other	4 (13)
Multiple osteoarticular sites	9 (28)
Other sites of infection	
Lung	19 (59)
Skin/soft tissue	8 (25)
Meningitis	2 (6)
Hardware involvement	3 (9)
Presenting symptoms	
Pain	28 (88)
Joint swelling	11 (34)
Fever	5 (16)
Night sweats	3 (9)
Weight loss	4 (13)
Pulmonary symptoms	4 (13)
Skin lesion/wound	4 (13)
Other	11 (34)
Multiple symptoms	23 (72)
Symptom duration before diagnosis, median (IQR), mo	5 (2–12)
Laboratory tests, median (IQR)	
CF titer at diagnosis	64 (12–128)
CF titer maximum	64 (24–256)
Erythrocyte sedimentation rate, mm/h	33.0 (14.0–85.0)
C-reactive protein, mg/dL	1.9 (0.9–9.7)

The most frequently involved skeletal sites were the spine (n = 15, 47%) and knee (n = 9, 28%), and 9 (28%) had multiple osteoarticular sites involved (Table 1). Detailed characteristics of the 2 most common sites of infection, spine and knee, are presented in Supplementary Tables 1 and 2. Of the 9 cases with knee infection, 8 (87%) had a confirmed diagnosis of native septic arthritis by positive synovial culture or pathology for *Coccidioides*, while 1 (11%) had confirmed intraarticular osteomyelitis by positive culture or pathology on bone without positive synovial culture or pathology. All 8 cases of septic arthritis also had microbiologic or radiographic findings suggestive of intraarticular bony involvement. Knee aspiration was performed in 3 patients where nucleated cell count ranged from 8775 to 132 500 cells/µL, percent neutrophils ranged from 49% to 98%, percent lymphocytes ranged from 1% to 25%, percent monocytes ranged from 0% to 26%, and percent eosinophils ranged from 0% to 1%. Cervical spine was the most common spinal level involved, seen in 9 of 15 (60%) cases, followed by lumbar with 7 (47%) cases. Multiple spinal regions were involved in 7 (47%) cases.

X-ray results were available in 17 (53%) patients, while magnetic resonance imaging (MRI) was available in 27 (84%) (Table 2). Bony erosion (24%) and degenerative changes (24%) were the most common findings reported on x-ray, while bone marrow edema or enhancement (81%) and soft tissue or synovial enhancement (66%) were the most common findings reported on MRI. In addition to x-ray and MRI, computed tomography scan was available in 6 (19%) patients and nuclear medicine study was available in 7 (22%). Representative images are presented as Supplementary Figures 1–3.

**Table 2.** Summary X-ray and MRI Findings

	Total n = 32, No. (%)
X-ray	17 (53)
Bony erosion	4 (24)
Degenerative changes/joint space narrowing	4 (24)
Joint effusion	3 (18)
Lucency	2 (12)
Mass/lytic lesion	2 (12)
Periosteal reaction	1 (6)
MRI	27 (84)
Bone marrow edema/enhancement	21 (81)
Soft tissue/synovial enhancement	19 (70)
Bony erosion	15 (56)
Abscess/fluid collection	11 (41)
Mass/lytic lesion	10 (37)
Joint effusion	9 (33)

### Treatment and Outcomes

Antifungal therapy most commonly consisted of itraconazole (n = 16, 50%), posaconazole (n = 8, 25%), and fluconazole (n = 6, 19%) (Table 3). Liposomal amphotericin B (n = 1, 3%) and voriconazole (n = 1, 3%) were used less frequently, while 1 patient (3%) received combination therapy. The median treatment duration (IQR) was 45 (26–90) months. Surgical intervention was performed in 24 patients (75%), with a median (IQR) of 3 (1–4) procedures. Local antifungal (amphotericin) was administered in 13 patients (34%).

**Table 3.** Summary of Treatment and Outcomes

	Total n = 32, No. (%)
Predominant antifungal therapy	
Liposomal amphotericin B	1 (3)
Fluconazole 400 mg	2 (6)
Fluconazole 800 mg	3 (9)
Fluconazole >800 mg	1 (3)
Itraconazole^a^	16 (50)
Posaconazole^a^	8 (25)
Voriconazole^a^	1 (3)
Isavuconazole^a^	1 (3)
Combination	1 (3)
Antifungal duration, median (IQR), mo	45.0 (26.4–89.8)
Surgical procedures	
Underwent surgery	24 (75)
No. of surgeries, median (IQR)	3 (1–4)
Local antifungal delivery used	11 (34)
Treatment failure	
Progression	8 (25)
Relapse	8 (25)
Outcome at last follow-up	
Off antifungals	6 (19)
On antifungal for treatment of ongoing signs/symptoms	17 (53)
On antifungal for suppression with no signs/symptoms	9 (28)

^a^Drug dose was based on therapeutic drug monitoring.

Progression while on antifungal therapy occurred in 8 patients (25%), with a median time to progression of 9 months, while relapse after stopping antifungals was documented in 8 patients (25%). (Table 3) One patient experienced both progression and relapse. Detailed information on patients who experienced progression is presented in Supplementary Table 3. At the last infectious disease clinic visit, 9 patients (28%) remained on antifungal therapy for suppression without active symptoms, 17 (53%) were on treatment for ongoing symptoms, and only 6 (19%) had successfully discontinued antifungal therapy. Median follow-up (IQR) was 84 (47–127) months.

### Risk Factors for Adverse Outcomes

Among the 32 patients, 15 (47%) experienced progression and/or relapse, while 17 (53%) did not (Table 4). On univariable analysis, knee infection (OR, 18.29; 95% CI, 1.91–175.35; *P* = .01) and multiple sites of infection (OR, 6.56; 95% CI, 1.10–39.32; *P* = .05) were significantly associated with relapse and/or progression while spine infection (OR, 0.20; 95% CI 0.44–0.90; *P* = .04) was associated with decreased rates of relapse or progression. Prolonged symptom duration before antifungal initiation was also associated with progression and/or relapse (OR, 1.07; 95% CI, 0.10–1.14; *P* = .04). There were no differences in outcomes based on CF titers, antifungal agents used, and whether the patient underwent surgery.

**Table 4.** Risk Factors of Disease Progression and Relapse

	No Progression or Relapse	With Progression or Relapse	*P* Value	
	n = 17	n = 15		OR (95% CI)
Age	53.8 (40.5–70.0)	46.0 (32.1–55.1)	.19	
Gender	16 (94)	13 (87)	.59	
Non-Hispanic Black	4 (24)	6 (40)	.45	
Comorbid conditions				
Diabetes mellitus	3 (18)	7 (47)	.13	
Immunosuppressed	2 (12)	2 (13)	1	
Involved bone/joint				
Spine	11 (65)	4 (27)	.042	0.20 (0.44–0.90)
Wrist/hand	1 (6)	2 (13)	.59	
Elbow	0 (0)	1 (7)	.47	
Shoulder	0 (0)	5 (33)	.015	NA
Hip	1 (6)	2 (13)	.59	
Knee	1 (6)	8 (53)	.005	18.29 (1.91–175.35)
Foot/ankle	1 (6)	1 (7)	1.00	
Long bone lower extremity	1 (6)	3 (20)	.32	
other	4 (24)	0 (0)	.10	
Multiple sites	2 (12)	7 (47)	.049	6.56 (1.10–39.32)
Hardware involvement	0 (0)	3 (20)	.092	
Symptom duration, median (IQR), mo	4 (1–6)	7 (2–36)	.044	1.07 (0.10–1.14)
Laboratory tests				
CF titer at diagnosis	32 (8–64)	64 (32–128)	.17	
CF titer maximum	32 (8–128)	64 (32–256)	.15	
Erythrocyte sedimentation rate, mm/h	41.0 (22.0–86.0)	32.0 (13.5–64.5)	.46	
C-reactive protein, mg/dL	1.9 (0.5–7.5)	2.3 (0.9–16.8)	.60	
Antifungal therapy				
Liposomal amphotericin B	0 (0)	1 (7)	.47	
Fluconazole	3 (18)	3 (20)	1.00	
Itraconazole	8 (47)	8 (53)	1.00	
Other triazole	6 (35)	4 (27)	.71	
Combination	0 (0)	1 (7)	.47	
Underwent surgery	9 (53)	13 (87)	.060	

### Characteristics of Patients who Came Off Antifungal Treatment

Characteristics of patients who completed an antifungal course are summarized in Table 5. In this subgroup, median treatment duration (IQR) was 27 (16–32) months before stopping treatment. Of the 13 patients who stopped treatment, 8 (62%) experienced relapse with a median time to relapse (IQR) of 17 (5–56) months from time of stopping antifungals. Of the 8 patients who experienced relapse, 4 had negative CF titers at the end of therapy, and 7 remained on antifungals through their last clinic follow-up after resuming antifungal treatment. For all 5 patients who did not experience relapse, CF titers were still positive when antifungals were stopped. The decision to stop antifungal treatment was largely based on clinical evaluation of patient symptoms and CF titer. Imaging and inflammatory markers were specifically reviewed at the time of discontinuation in 4 patients.

**Table 5.** Summary of Patients who Came Off Antifungal Treatment

Relapse	Age/Race	Site of Infection	Comorbidity	Max CF Titer	CF Titer at End of Antifungal Therapy	No. of Surgeries	Antifungal Before Relapse	Antifungal After Relapse	Antifungal Duration, mo	Time to Relapse From End of Antifungal Therapy, mo	Total Follow-up Duration, mo	Final Outcome
Yes	46 Asian	Knee	Known prior diagnosis	1:64	Negative	1	Itraconazole	Itraconazole	58	17	125	On suppression with no signs/symptoms of infection
Yes	66 Other	Knee, shoulder, long bone lower extremity	Known prior diagnosis	1:64	Negative	4	Fluconazole 400 mg	Itraconazole -> fluconazole 600 mg	41	1	84	On suppression with no signs/symptoms of infection
Yes	55 Hispanic	Knee	Known prior diagnosis, diabetes mellitus	1:16	1:4	1	Voriconazole	Voriconazole	16	7	163	On antifungal for ongoing signs/symptoms of infection
Yes	45 Non-Hispanic White	Knee	None	1:256	1:16	1	Itraconazole	Fluconazole 800 mg -> 400 mg	11	43	141	On antifungal for ongoing signs/symptoms of infection
Yes	38 Non-Hispanic White	Wrist/hand	Known prior diagnosis	1:32	Negative	3	Itraconazole	Itraconazole	26	87	132	Off antifungal
Yes	55 Non-Hispanic White	Hip	None	1:256	1:16	4	Itraconazole	Posaconazole	12	16	157	On antifungal for ongoing signs/symptoms of infection
Yes	31 Non-Hispanic Black	Spine, hip	Known prior diagnosis	1:64	1:8	2	Itraconazole	Posaconazole	33	2	61	On antifungal for ongoing signs/symptoms of infection
Yes	41 Hispanic	Shoulder, foot/ankle	Known prior diagnosis, diabetes mellitus, immunosuppressed	1:32	Negative	3	Itraconazole	Itraconazole -> posaconazole	13	67	130	On antifungal for ongoing signs/symptoms of infection
No	20 Other	Spine, long bone lower extremity	None	1:512	1:8	0	Itraconazole		32		50	Off antifungal
No	21 Asian	Pelvis	None	1:64	1:2	0	Posaconazole		19		45	Off antifungal
No	45 Non-Hispanic Black	Spine	Known prior diagnosis	1:64	1:2	1	Posaconazole		30		57	Off antifungal
No	74 Non-Hispanic White	Hip	Diabetes mellitus	1:4	1:2	2	Posaconazole		154		155	Off antifungal
No	84 Hispanic	Foot/ankle	None	1:32	1:32	3	Fluconazole 800 mg		27		36	Off antifungal

## DISCUSSION

This study highlights the varied clinical presentations and long-term outcomes of osteoarticular coccidioidomycosis, offering a more detailed understanding of the disease in adults. The predominance of male patients, frequent spinal involvement, and high rate of surgical intervention align with existing literature [2, 4, 7]. Our findings significantly add to the literature base by identifying certain risk factors for treatment failure and providing robust patient-level data to better illustrate variations in treatment approach and outcomes.

In concordance with prior research, we found that the spine and knee were the most common sites of infection, with multisite infection also relatively frequent, signifying greater disease severity [2, 3, 7–9]. We identified knee and multisite infection as risk factors for treatment failure, whereas spinal infections had the most favorable prognosis. These observations suggest that skeletal site may carry distinct prognostic implications that warrant closer attention in future studies.

Diagnosis of osteoarticular coccidioidomycosis is often delayed because pain and swelling resemble more common conditions such as osteoarthritis [10]. Reviews have consistently highlighted these diagnostic challenges, particularly outside endemic regions where suspicion is lower [2, 10]. In our cohort, the median duration of symptoms before antifungal therapy was 5 months, reflecting the impact of such delays. Although immunosuppression and diabetes are recognized risk factors for dissemination, we found that only 16% of patients were immunocompromised and 33% had diabetes [11]. These findings demonstrate that classic risk factors are important but not sufficient to capture disease burden, reinforcing the need for early clinical suspicion in any patient with unexplained musculoskeletal symptoms in areas where coccidioidomycosis is endemic.

The management of osteoarticular coccidioidomycosis continues to rely on antifungal therapy, with azoles as the mainstay and amphotericin B reserved for severe or disseminated disease [4, 5]. Randomized trial data comparing azoles with amphotericin are lacking; however, a subgroup analysis of a double-blind randomized controlled trial comparing fluconazole and itraconazole for treatment of coccidioidomycosis showed that itraconazole may be more effective in skeletal disease at 8 or 12 months of therapy [12]. Itraconazole was the most commonly used antifungal agent in our cohort, but outcomes did not differ significantly across antifungal therapies. Further studies are needed to better characterize the optimal antifungal agent, particularly given that data are lacking for newer triazoles and with novel antifungal agents such as olorofim emerging as an alternative treatment option [13].

Antifungal therapy alone, however, is often insufficient, and surgical debridement, sometimes combined with local antifungal delivery, is often employed for disease control [4, 5]. Our surgical rate of 75% is comparable to prior reports in both pediatric patients and adults where about 81% of patients underwent operative intervention [9, 14]. At our institution, osteoarticular coccidioidomycosis management relied on a close partnership between orthopedic surgery and infectious disease specialists. This collaboration facilitated coordinated planning and an aggressive surgical approach that frequently combined extensive debridement with local antifungal placement, often with multiple planned repeat debridement and antifungal delivery device exchange. The median number of surgeries in our cohort was higher than often reported, likely reflecting this proactive strategy. The role of surgery remains unclear, however, as in our cohort patients who underwent surgery trended toward higher rates of progression or relapse, likely reflecting selection bias.

Despite prolonged therapy, high rates of treatment failure are noted in disseminated coccidioidomycosis, as demonstrated by our results where we saw progression or relapse in nearly half of our cohort. While prior studies have also demonstrated high treatment failure rates, they have not delineated specific patient characteristics associated with relapse or progression [8, 14]. To our knowledge, this is the first study to identify multisite involvement, knee infection, and prolonged symptom duration before treatment as risk factors for adverse outcomes. Interestingly, spinal involvement was associated with more favorable outcomes, a finding not previously described. We postulate that this may reflect more effective antifungal delivery due to the high vascular supply of the spine compared with joints of more complex anatomy. Conversely, knee infection was associated higher rates of treatment failure. This may be due to issues with source control as all of our cases of knee septic arthritis likely had concurrent intraarticular bony involvement, which may have made surgical source control challenging to achieve.

Determining when to stop antifungal therapy remains one of the most difficult aspects of managing osteoarticular coccidioidomycosis. Clinicians often rely on clinical and laboratory data, including symptom improvement and CF titer trends, but there is no standardized method established regarding when to stop therapy [5]. McHardy et al. studied serologic responses in disseminated disease and found that 31% of patients showed no meaningful decline in CF titers despite prolonged treatment, even when they were clinically stable. Some patients maintained high titers without evidence of worsening disease, while others relapsed after titers became negative [15]. This disconnect shows that quantitative CF titers do not reliably reflect disease activity.

Our findings illustrate the same problems with understanding for whom it is appropriate to stop systemic antifungal therapy. In the subgroup of patients who stopped antifungal treatment, relapse occurred in patients who had negative CF titers at the end of therapy. Furthermore, of the patients who did not experience relapse, all had positive CF titers ranging from 1:2 to 1:32 at the time of antifungal cessation. These results suggest that CF testing should be considered supportive evidence of improvement rather than a definitive measure of disease activity. They also highlight the need for standardized treatment guidelines or risk assessment scores to help guide therapy duration and decision regarding antifungal cessation. After discontinuing therapy, close patient follow-up is essential to reliably distinguish controlled infection from relapse and to guide further management.

This study has a number of limitations. First, its retrospective single-center design restricts generalizability. Second, we were unable to reliably assess treatment adherence, which may influence relapse risk. Therapeutic drug monitoring was performed in our cohort, but the drug level goal was not consistently documented, and the frequency with which to measure drug levels varied by provider. Third, the sample size precluded us from performing a more robust statistical analysis, and therefore confounding variables were not controlled for. Even so, the strength of this work lies in the detailed patient-level data and long follow-up duration.

## CONCLUSIONS

Osteoarticular coccidioidomycosis in adults is an uncommon but devastating manifestation of disseminated coccidioidomycosis, frequently affecting the spine and knee. Our study highlights that the involvement of the knee joint and multisite involvement may be an adverse prognostic marker. Early recognition and multidisciplinary management are essential to optimize outcomes. Further research is needed to define optimal medical and surgical treatment strategies, identify high-risk patients, and develop novel antifungal agents and biomarkers. These steps are critical to improve long-term outcomes in osteoarticular coccidioidomycosis, where relapse and progression remain common.

## Supplementary Material

ofag103_Supplementary_Data

## Supplementary Data

Supplementary materials are available at *Open Forum Infectious Diseases* online. Consisting of data provided by the authors to benefit the reader, the posted materials are not copyedited and are the sole responsibility of the authors, so questions or comments should be addressed to the corresponding author.
