# Supplementary material for: Osteoarticular Coccidioidomycosis in California: A Single-Center Experience
Source: Open Forum Infect Dis. 2026 Feb 24;13(3):ofag103. doi: 10.1093/ofid/ofag103 (PMC12978523; doi:10.1093/ofid/ofag103)
Supplement: ofag103_Supplementary_Data [file ofag103_supplementary_data.zip › Supplemental figures.pptx]

## Slide 1
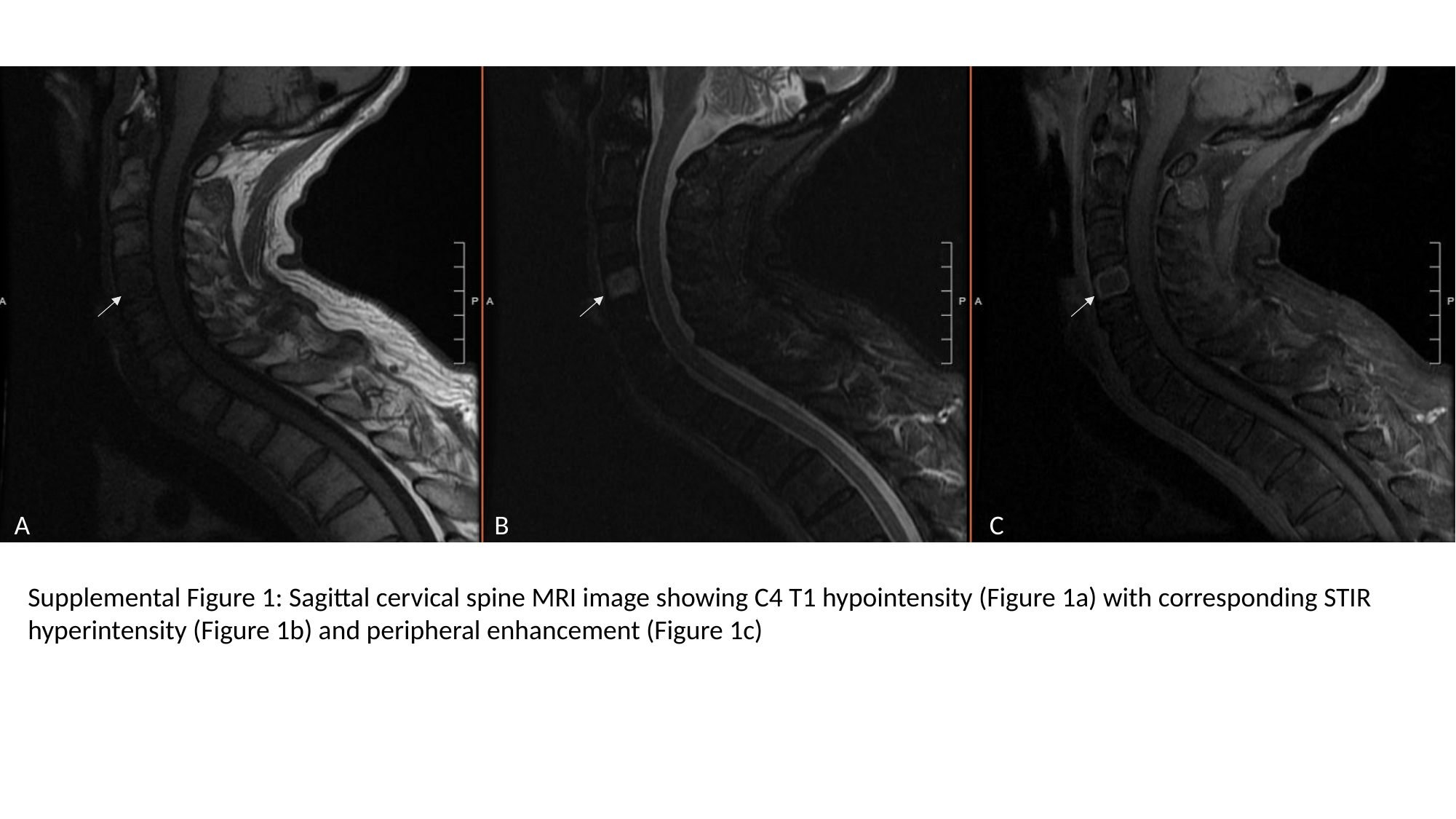

A
C
B
Supplemental Figure 1: Sagittal cervical spine MRI image showing C4 T1 hypointensity (Figure 1a) with corresponding STIR hyperintensity (Figure 1b) and peripheral enhancement (Figure 1c)

## Slide 2
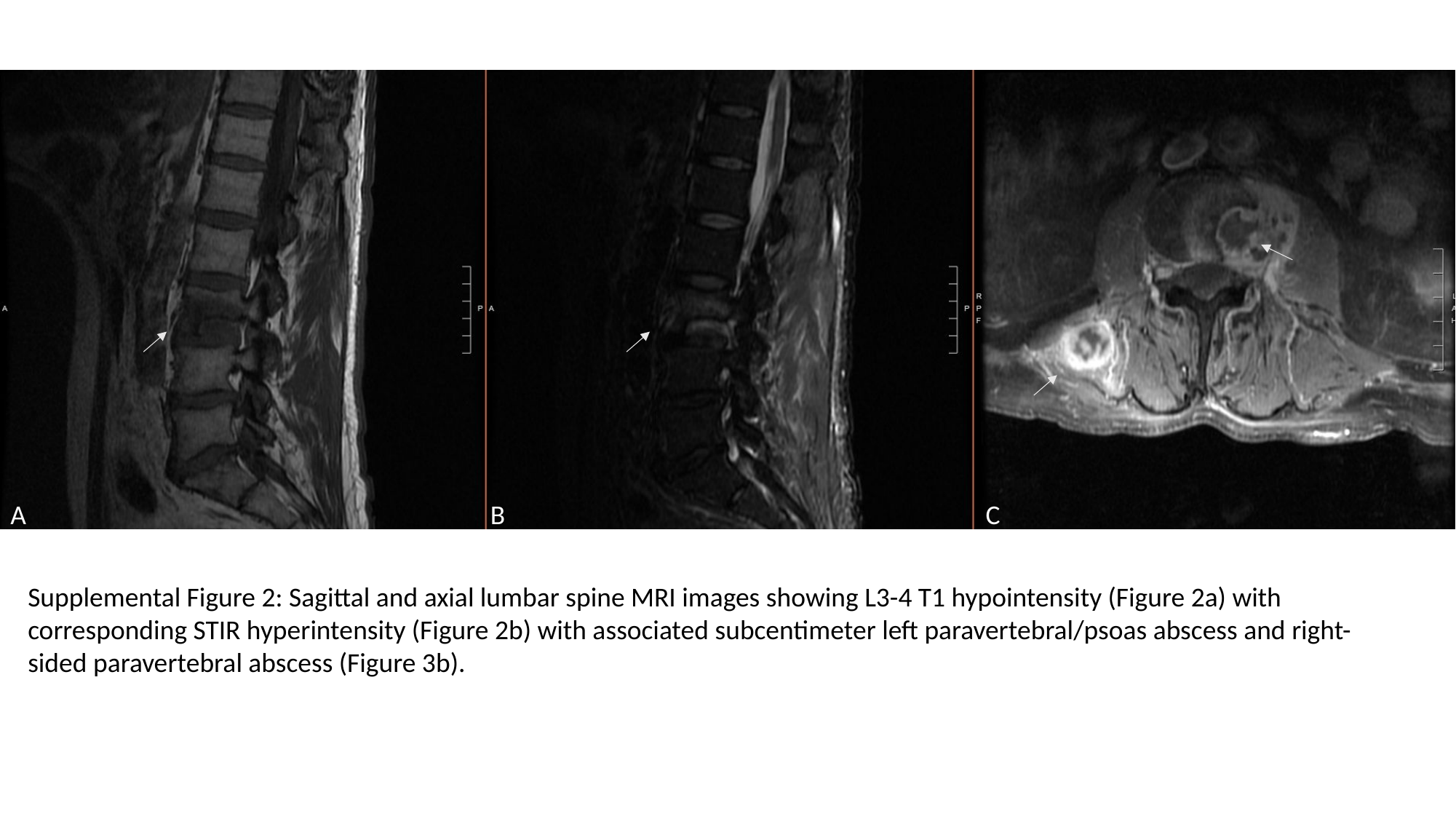

A
C
B
Supplemental Figure 2: Sagittal and axial lumbar spine MRI images showing L3-4 T1 hypointensity (Figure 2a) with corresponding STIR hyperintensity (Figure 2b) with associated subcentimeter left paravertebral/psoas abscess and right-sided paravertebral abscess (Figure 3b).

## Slide 3
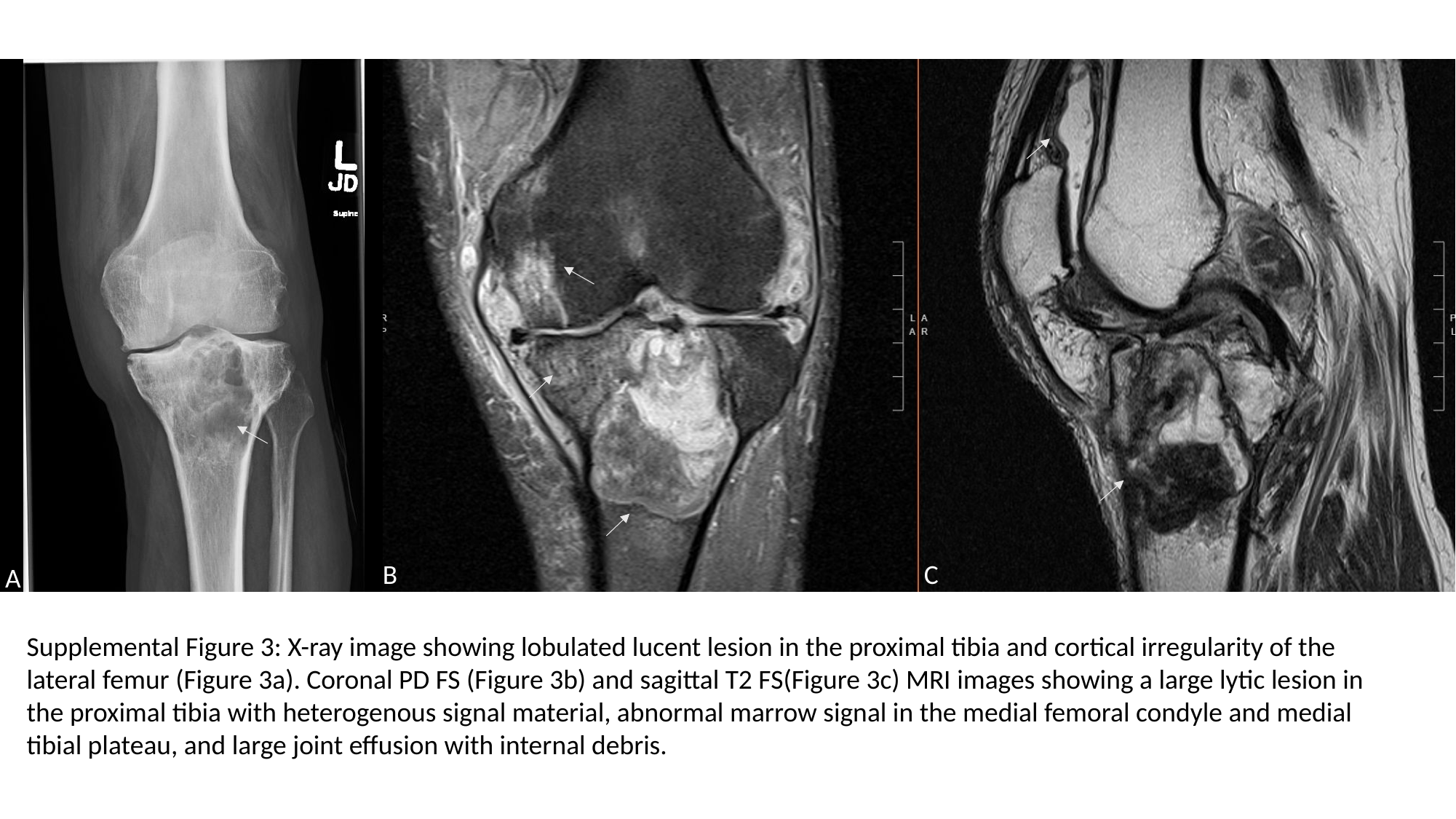

B
C
A
Supplemental Figure 3: X-ray image showing lobulated lucent lesion in the proximal tibia and cortical irregularity of the lateral femur (Figure 3a). Coronal PD FS (Figure 3b) and sagittal T2 FS(Figure 3c) MRI images showing a large lytic lesion in the proximal tibia with heterogenous signal material, abnormal marrow signal in the medial femoral condyle and medial tibial plateau, and large joint effusion with internal debris.
