# Supplementary material for: Osteoarticular Coccidioidomycosis in California: A Single-Center Experience
Source: Open Forum Infect Dis. 2026 Feb 24;13(3):ofag103. doi: 10.1093/ofid/ofag103 (PMC12978523; doi:10.1093/ofid/ofag103)
Supplement: ofag103_Supplementary_Data [file ofag103_supplementary_data.zip › Supplemental Tables.docx]

Supplemental Table 1: Characteristics of patients with knee infection

|  | Total  N=9 n (%) |
| --- | --- |
| **Infection type** |  |
| Native septic arthritis | 8 (89%) |
| Intraarticular osteomyelitis | 1 (11%) |
| **Hardware involvement** | 0 (%) |
| **Concurrent infection** |  |
| Other osteoarticular site | 3 (33%) |
| Meningitis | 0 (%) |
| **Symptoms** |  |
| Pain | 9 (100%) |
| Joint swelling | 8 (89%) |
| Fever | 1 (11%) |
| Night sweats | 1 (11%) |
| Weight loss | 1 (11%) |
| Pulmonary symptom | 0 (0%) |
| Sinus tract | 1 (11%) |
| **Imaging findings** |  |
| Bone marrow edema/enhancement | 8 (89%) |
| Soft tissue/synovial enhancement | 4 (44%) |
| Bony erosion | 4 (44%) |
| Lytic/mass lesion | 3 (33%) |
| Abscess/fluid collection | 1 (11%) |
| Joint effusion | 7 (78%) |
| **Surgical procedures** |  |
| Underwent surgery | 9 (100%) |
| Debridement (native bone/joint) | 9 (100%) |
| Hardware placed | 1 (11%) |
| Hardware exchanged | 1 (11%) |
| Amputation | 1 (11%) |
| Number of surgeries (median, IQR) | 3 (1-7.5) |

Supplemental Table 2: Characteristics of patients with spinal infection

|  | Total  N=15 n (%) |
| --- | --- |
| **Spinal level** |  |
| Cervical | 9 (60%) |
| Thoracic | 5 (33%) |
| Lumbar | 7 (47%) |
| Sacral | 4 (27%) |
| Multiple spinal regions | 7 (47%) |
| **Extent of infection** |  |
| Vertebral osteomyelitis | 14 (93%) |
| Discitis | 5 (33%) |
| Paraspinal abscess | 8 (53%) |
| Epidural extension | 8 (53%) |
| **Hardware involvement** | 1 (7%) |
| **Concurrent infection** |  |
| Other osteoarticular site | 6 (40%) |
| Meningitis | 2 (13%) |
| **Symptoms** |  |
| Pain | 13 (87%) |
| Fever | 4 (27%) |
| Night sweats | 1 (7%) |
| Weight loss | 3 (20%) |
| Pulmonary symptom | 4 (27%) |
| **Imaging findings** |  |
| Bone marrow edema/enhancement | 11 (73%) |
| Soft tissue/synovial enhancement | 11 (73%) |
| Bony erosion | 8 (53%) |
| Lytic/mass lesion | 4 (27%) |
| Abscess/fluid collection | 7 (47%) |
| **Surgical procedures** |  |
| Underwent surgery | 7 (47%) |
| Debridement (native bone/joint) | 6 (86%) |
| Hardware placed | 4 (57%) |
| Hardware exchanged | 1 (14%) |
| Number of surgeries (median, IQR) | 1 (1,2) |

Supplemental Table 3: Summary of patients who experienced disease progression

| Age/  Race | Site of infection | Comorbidity | Max CF titer | Number of surgeries | Antifungal prior to progression | Antifungal after progression | Time to progress from start of antifungal therapy | Total antifungal duration (months) | Total follow up duration (months) | Final outcome |
| --- | --- | --- | --- | --- | --- | --- | --- | --- | --- | --- |
| 46  Asian | knee | known prior diagnosis | 1:64 | 1 | fluconazole 200mg | fluconazole 600mg -> itraconazole | 46 | 58* | 125 | On suppression with no signs/symptoms of infection |
| 65  Other | spine, wrist/hand, shoulder, knee, long bone lower extremity | none | 1:128 | 12 | fluconazole 1200mg | fluconazole 1200mg -> fluconazole 800mg -> fluconazole 600mg -> fluconazole 400mg | 10 | 91 | 91 | On suppression with no signs/symptoms of infection |
| 26  Non-Hispanic White | shoulder | immunosuppressed | 1:64 | 1 | liposomal amphothericin, voriconazole | liposomal amphothericin+voriconazole -> voriconazole -> posaconazole | 0.5 | 98 | 98 | On suppression with no signs/symptoms of infection |
| 19  Hispanic | knee, long bone lower extremity | none | 1:1024 | 9 | itraconazole | fluconazole 400mg -> isavuconazole | 1 | 33 | 33 | On antifungal for ongoing signs/symptoms of infection |
| 47  Non-Hispanic White | knee | none | 1:16 | 3 | fluconazole 800mg | itraconazole -> fluconazole 800 mg-> fluconazole 400mg | 10 | 46 | 46 | On antifungal for ongoing signs/symptoms of infection |
| 55  Hispanic | spine, shoulder | diabetes mellitus, known prior diagnosis | 1:256 | 7 | itraconazole | posaconazole | 93 | 96 | 96 | On antifungal for ongoing signs/symptoms of infection |
| 32  Hispanic | spine, elbow | known prior diagnosis | 1:256 | 7 | liposomal amphothericin | liposomal amphothericin, posaconazole | 3 | 110 | 110 | On antifungal for ongoing signs/symptoms of infection |
| 46  Non-Hispanic White | knee | known prior diagnosis | 1:128 | 6 | itraconazole | posaconazole | 10 | 54 | 54 | On antifungal for ongoing signs/symptoms of infection |

*This patient also subsequently experienced relapse (first patient in Table 5). The antifungal duration presented is the duration of antifungal prior to relapse.
